# Prediction of problem gambling by demographics, gaming behavior and psychological correlates among gacha gamers: A cross-sectional online survey in Chinese young adults

**DOI:** 10.3389/fpsyt.2022.940281

**Published:** 2022-08-05

**Authors:** Anson Chui Yan Tang, Paul Hong Lee, Simon Ching Lam, Summer Cho Ngan Siu, Carmen Jiawen Ye, Regina Lai-Tong Lee

**Affiliations:** ^1^Tung Wah College, Hong Kong, Hong Kong SAR, China; ^2^University of Leicester, Leicester, United Kingdom; ^3^Hong Kong Metropolitan University, Hong Kong, Hong Kong SAR, China; ^4^Lingnan University, Hong Kong, Hong Kong SAR, China; ^5^The Chinese University of Hong Kong, Hong Kong, Hong Kong SAR, China

**Keywords:** problem gambling, gacha, psychological distress, video game microtransaction, Chinese young adults

## Abstract

**Objective:**

The objective of this study is to explore the association of problem gambling with demographics, psychological distress, and gaming behavior in young adult gacha gamers in Hong Kong.

**Materials and methods:**

Cross-sectional data was collected in the first and fifth waves of COVID-19 pandemic in Hong Kong online. Participants who aged 18–25 years and had been playing gacha games over the past 12 months were recruited. Stepwise multiple regression was used to explore the association among risk of problem gambling, gaming behavior, participation in gaming activities and psychological distress. A two-sided p-value <0.05 was considered as statistical significance.

**Results:**

Three hundred and thirty-seven completed questionnaires were received with no missing data. 34.7% (*n* = 117) of the participants had non/low-risk of problem gambling. About 40% (*n* = 136) of them had moderate-risk and the remaining 25% (*n* = 84) were at high risk of problem gambling. A higher proportion of female participants (78.6%) were found in high-risk group as compared to 39.7% and 55.6% only in the non/low-risk and moderate-risk groups, respectively. The regression model (*R*^2^ = 0.513, *F* = 71.895, *p* < 0.001) showed that 51.3% of the variance of the total problem gambling score could be explained by stress, anxiety, monthly expenses on gacha purchases, number of motives for gacha purchase and number of gambling activities engaged.

**Conclusion:**

The present study provides empirical evidence to support the association between problem gambling and microtransaction especially for gacha which is the most popular type of video game microtransaction in Asia. The established regression model suggests that gacha gamers with higher risk of problem gambling tend to have greater stress, higher anxiety level, spend more on gacha purchase, have more motives for gacha purchases and engage in more gambling activities. In contrast to the extant literature, higher proportion of female participants in high-risk group indicates that female gacha gamers are also at very high risk of becoming problem gamblers.

## Introduction

Microtransaction is the mainstream to monetize gaming activities in gaming industry. As of 2018, 50–91% of the highest grossing mobile games in United Kingdom, Australia and China contain microtransactions (1–3). Gacha and loot box are the two common forms of video game microtransaction dominant in Asian and Western regions, respectively. Both share a similarity that game players have to pay for the chance of obtaining virtual items that could advance the game progress or enhance character abilities (4). In Asian region, gacha is currently predominant in the mobile game market especially in Freemium (i.e., free-to-play) games. It replicates the feature of capsule toy vending machines popularized in Japan where gacha gamers pay to obtain in-game randomized virtual items such as weapons and costumes that can enhance the power and appearance of their game avatar. The virtual items’ degree of rarity is determined and controlled by gaming companies (4, 5). The popularity of gacha can be glimpsed from the market share and rapidly growing revenue of mobile game market. The domestic Chinese game market was projected to reach US$42billion in revenue by 2022 and over one quarter of the global mobile gaming revenue comes from China (6). In 2020, 78% of the total revenue of digital games spending came from Freemium games and 58% of the digital game spending occurred on smartphones (7). The total revenue of Hong Kong mobile game market increased dramatically from HK$15 million in 2010 to HK$44.6 million in 2014 (8). The increased possession of smartphones facilitate participation in gacha games. A report showed that 86.3% of Hong Kong gamers used smartphone most frequently as their primary gaming device (9). It is estimated that among the two million game players in Hong Kong, 15% of them play gacha games (10).

The chance-based item-purchasing process of video game microtransaction, which is closely connected to the opportunity of winning games, is compelling to gamers and could promote gambling among game players (11). Social media has frequently reported the excessive spending on gacha games in adolescents and young adults which result in gambling and emotional problems (12). About 30% of gacha game players are students with no income or monthly income less than HK$5,000 (12). They spend from HK$2,000 to up to HK$10,000 per month (13). Gambling cases caused by uncontrollable gacha purchases have increased drastically. The number of reported gambling cases due to gacha purchases in the first quarter of 2019 was 70% of the total cases in the preceding year (14).

Adolescents and young adults who participate in video game microtransaction have been identified as the most susceptible group for problem gambling. Empirical evidence has shown that the prevalence of problem gambling in adolescents and young adults is higher than the other age groups (15). Studies in Europe and United States have shown that among the 63.82% of adolescents and young adults who participated in gambling activities, 4–7% of them manifested serious gambling symptoms and 10–15% of them were at risk for developing serious gambling problem (16). In addition, they are the dominant consumers for video games, 50–60% of which contain microtransactions to monetize gaming activities (1–3, 17, 18). And 62.6–94.9% of these mobile games were deemed suitable for adolescents aged 12 years or above (19). Relatedly, recent statistics for loot box showed that 36%–46% of video game players make in-game purchases (20, 21). This phenomenon was believed to be amplified by the COVID-19 locally and internationally. Claesdotter-Knutsson et al. (22) reported that 16–39 years people showed increased engagement in loot box gaming during COVID-19 pandemic in Sweden. Similar statistics were found in Hong Kong. During the COVID-19 pandemic, nearly all normal economic activities were suspended. People worked and studied online at home. Adolescents and young adults were forced to stay at home most of the time with very limited outdoor activities but playing games to kill time (13). A recent local report about gacha gaming behavior in adolescents and adults showed that over half of them spend 3 h or more to play gacha games every day (13). These findings are alarming to stakeholders including social and healthcare professionals as the gambling behavior developed at a young age is likely to continue and be amplified, eventually resulting some form of gambling disorder (23). Thus, there is a pressing need to recognize young adult gacha game players predisposition to problem gambling.

Gambling disorder is a non-substance-related mental disorder listed in the Fifth Edition of the Diagnostic and Statistical Manual of Mental Disorders (24). It is currently defined as persistent and recurrent engagement in gambling behavior that leads to clinically significant impairment or distress (24). Recent studies have shown that video game microtransactions are associated with a greater risk of problem gambling (25–30). Systematic reviews, meta-analyses, and cross-sectional studies have reported a significant relationship between problem gambling and video game microtransaction, mainly through loot box (31–37) but rarely with gacha. For example, Garea et al. (36) and Delfabbro and Cairns (30) found a significant small-to-moderate positive relationship between loot box spending and gambling symptoms. One cross-sectional study reported a moderate-to-large effect for the relationship between loot box purchase and problem gambling in a sample of older adults (18). While many studies have recruited adolescent samples, e.g., Kristiansen and Severin (32) and Zendle et al. (18), only a few have focused on young adults (38, 39).

Although spending on video game microtransactions is often the major focus in the extant literature concerning problem gambling in video game microtransactions, it appears that it is not the only predisposing factor for problem gambling among gamers. Some researchers have attempted to determine problem gambling’s relationship with other gaming-related behavior, such as the symptoms of problem gaming and the motives behind video game microtransactions. Recent cross-sectional studies demonstrated a relationship between problem gaming and problem gambling such as King et al. (39) and Drummond et al. (40), with the former (39) revealing that problem gamblers are 5.62 times more likely to engage in problem gaming. A handful of studies identified loot box game players’ motives for conducting video game microtransactions. Motives that were positively correlated with loot box spending were mostly connected to social recognition, excitement, obtaining specific items/characters, gaining privileges to progress in a game, among others (18, 31, 37). Enhancing self-esteem was also found to be positively correlated with loot box purchase (37).

In addition, researchers examined the risk of problem gambling in terms of gamers’ psychological wellbeing. Studies on loot box spending found that both positive and negative moods, as well as psychological distress were positively associated with loot box spending (37). Depression and anxiety were often reported to be associated with more loot box spending as well as symptoms of problem gaming and problem gambling (35, 41–43). Behavioral factors such as coping strategies, inattention, and conduct problems were also associated with more in-game item purchases and symptoms of problem gaming and gambling (42, 44). Furthermore, male gamers were found, by and large, to spend more on loot box as compared to female gamers (15, 25, 32, 37, 41, 45–47). Gamers with habits of online gambling and other gambling activities also had a higher risk of problem gambling (28, 48).

Apparently, whether gamers who participate in video game microtransactions will become problem gamblers depends on multiple factors. Some researchers have suggested that factors potentially contributing to the relationship between problem gambling and video game microtransactions should be considered altogether when exploring the relationship of problem gambling with video game microtransactions (30, 34). In addition, the studies conducted so far are mainly in Western samples, so the generalizability of these findings to Chinese samples is still under-investigated as studies have shown that there are significant cultural differences in gambling behavior (49, 50). Furthermore, as highlighted earlier, previous studies related to problem gambling in video game microtransactions focused on loot box but rarely gacha. The inherent differences between gacha and loot box may further limit the generalizability of the findings in previous studies. For example, gacha is mostly found in Freemium mobile games that are built on in-game item purchases and is the primary monetization strategy while loot box is often found in PC and console and is an additional monetization element besides selling the games themselves (4). In comparison to loot box, gacha has more varieties to attract gamers to pay for the games such as Box Gacha (virtual box of set items with known probabilities), Sugoroku Gacha (A gacha acts like a dice which then allowed the player to move on a board to unlock special items). Hence, the present study aimed to investigate the relationship between problem gambling and gacha with a broader spectrum by constructing a model to predict the risk of problem gambling according to demographics, gaming behavior, and psychological distress in gacha gamers.

The study objectives are as follows:

1. to explore the gacha gaming behavior and risk of problem gambling in Chinese young adults in Hong Kong and
2. to investigate the relationship between risk of problem gambling, gacha gaming behavior, demographics, and psychological distress in Chinese young adult gacha gamers.

## Materials and methods

### Study design and participants

An anonymous cross-sectional online survey was conducted in two periods: January to March of 2020 and January and March of 2022. The former period was the initial outbreak of COVID-19 in Hong Kong and the latter period was the fifth wave of COVID-19 in Hong Kong. Convenience sampling method was adopted to recruit eligible participants online. The project was promoted at three local universities/tertiary institutions as well as on local online gaming forums. Emails were sent to target students to complete the online survey in the participating universities/tertiary institutions. Students were encouraged to disseminate the online survey to their peers if they meet the selection criteria. The inclusion criteria were individuals (1) aged 18–25 years old (2) who had been playing gacha games over the past 12 months. The exclusion criterion was that individuals who cannot read Chinese. The online survey requested participants answer every question, so there was no missing data in the dataset. The minimum sample size required to achieve a study power of 0.8, alpha of 0.05, and medium effect size for association (*f*^2^ = 0.15) is 91 for five independent variables (51). A total of 337 participants were recruited over the aforementioned recruitment periods, thus giving the sufficient power for analysis.

Before commencing the online survey, the study objectives were clearly described to participants. All participants had to click the box “agree to participate in this study” to indicate their agreement to voluntarily participate in the study prior to starting the survey. Ethics approval of this study was obtained from the ethics committee of the corresponding author’s affiliated institution.

### Measures

#### Dependent variable

Risk of problem gambling was measured using the Problem Gambling Severity Index – Chinese version (PGSI-C). The PGSI-C is a 9-item questionnaire measuring one’s risk of problem gambling according to his/her gambling behavior and corresponding adverse consequences (52). It was translated from the original English version developed by Ferris and Wynne (53). Each item is rated via a 4-point Likert scale (0 = “never” to 3 = “almost always”). The total PGSI-C score is computed by summating all item ratings, with a score range of 0 to 27. The total PGSI-C score could also be categorized into three levels of gambling risk: (1) 0–2 = non/low-risk problem gambler; (2) 3–7 = moderate-risk problem gambler; and (3) ≥ 8 = high-risk problem gambler.

The PGSI-C was validated by Loo et al. in the Chinese adult population (52). The results showed good concurrent, discriminant, and predictive validity. Concurrent validity was measured by comparing the correlations of PGSI-C with other gambling scales such as South Oaks Gambling Screen, The Gambling Related Cognitions Scale, with *r* ranging from 0.34 to 0.53. Discriminant validity was investigated by examining whether PGSI-C discriminated between non-problem gamblers and possible problem gamblers. The discriminant function had significant difference between non-problem and possible problem gambling groups (Wilk’s Lambda = 0.60, χ^2^ = 197.68, df = 3, *p* < 0.001). Hierarchical multiple regression analysis was conducted to investigate the predictive validity of PGSI-C. The model explained 28.1% of the variance (adjusted *R*^2^ = 0.281, *p* < 0.001), with 26.5% variance explained by PGSI-C. In addition, Cronbach’s alpha of the PGSI-C was 0.77 (52). As PGSI-C was originally developed for screening general gambling activities, the authors had modified a few words on some items to fit the context of gacha games. Face validity and test-retest reliability were undertaken to ensure the modified version was comprehensible to the target population and sufficiently reliable. Twelve university students meeting the described selection criteria were recruited to complete the modified survey twice with a 2-week interval. The results demonstrated excellent test-retest reliability with an *r* of 0.954.

#### Independent variables

##### Psychological distress

Psychological distress was operationalized by the Depression, Anxiety and Stress 21 – Chinese version (DASS-21-C). The DASS-21-C was translated and validated by Gong et al. (54) based on the original English version established by Lovibond and Lovibond (55). The questionnaire consists of three subscales consisting of seven items each: Depression (items 3, 5, 10, 13, 16, 17, 21), Anxiety (items 2, 4, 7, 9, 15, 19, 20), and Stress (items 1, 6, 8, 11, 12, 14, 18). Respondents rate the items based on their experience in the past week on a 4-point Likert scale from 0 (“did not apply to me at all”) to 3 (“applied to me very much or most of the time”). Subscale scores are calculated by summing the ratings of the corresponding seven items and then multiplying by 2. The subscales range from 0 to 42 with higher scores indicating more symptoms. The DASS-21-C was found to have good psychometric properties. Cronbach’s alphas for depression, anxiety, and stress subscales were 0.896, 0.859, and 0.873, respectively, which indicate good internal consistency for each subscale (56). Moderate convergent validity of the Depression and Anxiety subscales was demonstrated via significant correlations with the Chinese Beck Depression Inventory (r = 0.64) and the Chinese State-Trait Anxiety Inventory (*r* = 0.41), respectively. Confirmatory factor analyses supported the original 3-factor model (non-normed fit index = 0.964, comparative fit index = 0.968 and root mean square of approximation = 0.079) (57). The test-retest reliabilities (*r*) of the depression, anxiety and stress subscales were 0.867, 0.917, and 0.913 respectively.

##### Gaming behavior

Gaming behavior was measured via three items: (1) average daily time spent playing gacha purchase in terms of hours; (2) average monthly expenses for gacha purchases; and (3) motives for gacha purchases. The first two items were ordinal data and referred to the experience in the past year. In contrast, motives for gacha purchases were nominal data. Participants could select more than one choice on the list provided.

##### Demographics and participation in gambling activities

Age, gender (male/female), and educational level (secondary school, diploma/certificate, associate degree/higher diploma, bachelor’s degree or above) were collected as they were reported to be associated with the risk of problem gambling in video game microtransactions. In addition, participation in gambling activities was included to measure the number and types of gambling activities (other than video game microtransactions) participants have engaged over the past year, such as football betting, lottery, horse racing, online gambling activities, etc. The gambling items were devised with reference to a local study about Hong Kong people’s participation in gambling activities (58). Age was continuous data. Gender and participation in gambling activities were nominal data and educational level was ordinal.

### Statistical analysis

The data analysis was performed using the SPSS version 26.0. Descriptive statistics were calculated to describe demographic variables, participation in gambling activities, gaming behavior, and psychological distress among the three levels of problem gamblers. Means and standard deviations or median and interquartile ranges were reported for continuous variables. Frequencies and percentages were computed for categorical and ordinal variables. To compare study variables with the three levels of problem gambling, the following tests were used: chi-squared test (nominal variables); one-way analysis of variance (ANOVA) or Kruskal-Wallis one-way ANOVA (continuous and ordinal variables) and adjusted by Bonferroni correction for multiple tests. Spearman’s rank correlation analysis was performed to test the correlation between the risk of problem gambling (i.e., total PGSI score) and other study variables. Stepwise multiple regression analysis was used to model the relationship among the total PGSI score, demographic variables, gaming behavior, participation in gambling activities, and psychological distress. A two-sided p value below 0.05 was considered statistically significant.

## Results

A total of 337 completed online surveys were returned with no missing data. Among all the participants, 34.7% (*n* = 117) had no or a low risk, 40.4% (*n* = 136) had a moderate risk, and 24.9% (*n* = 84) had a high-risk of problem gambling. There was a significant difference in age and gender among the three levels of problem gambling (*p* = 0.001 and <0.001, respectively). High-risk problem gamblers were significantly older than those in the non/low-risk and moderate-risk groups (*p* = 0.001 and 0.004, respectively). In addition, a higher proportion of females (78.6%) was found in the high-risk group compared to the other two groups, which ranged from approximately 39–55%.

All study variables were found to have significant differences among the three problem gambling groups. As compared with the non/low-risk and moderate-risk gamblers, high-risk gamblers were found to participate significantly more in gambling activities and have more motives for gacha purchase. In terms of daily time spent and monthly expenses for gacha purchases, high-risk gamblers spent significantly more time and money on gacha games compared to participants in the other two groups (*p* < 0.001). Approximately 60–80% of participants in the non/low-risk and moderate-risk problem gambling groups spent less than 4 h per day on playing gacha games. Conversely, about 55% of high-risk problem gamblers played gacha games for more than 4 h a day.

Regarding monthly spending on gacha purchases, 74–93% of non/low-risk and moderate-risk problem gamblers spent USD $50 or less on gacha games. In contrast, approximately 65% of high-risk problem gamblers spent over USD $50 every month on gacha purchases. Among them, 25% reported spending more than USD $200 per month. Furthermore, high-risk problem gamblers had significantly higher scores for the three DASS-21-C subscales compared to the other two groups (*p* = 0.000). Their median scores for depression, anxiety, and stress were 16, 13, and 18, respectively, thus indicating that high-risk problem gamblers exhibit moderate levels of depression, anxiety and stress, as suggested by Lovibond and Lovibond (55). Table 1 summarizes the comparison among the three risk levels of problem gambling in terms of demographic variables, gaming behavior, and psychological variables.

**TABLE 1:** Comparison among the three risk levels of problem gambling for demographic and independent variables (*N* = 337).

Study variables	Non/low-risk problem gambler (*n* = 117)	Moderate-risk problem gambler (*n* = 136)	High-risk problem gambler (*n* = 84)	*P*-value (a/b/c)
*Demographics*				
Age (years), M ± SD	21.44 ± 2.08	21.63 ± 2.16	22.57 ± 2.05	.001(1/.001/.004)^∧^
**Gender, n (%)**				
Male	52 (44.4)	82 (60.3)	18 (21.4)	<0.001*
Female	65 (55.6)	54 (39.7)	66 (78.6)	
**Educational level, n (%)**				
Secondary school	6 (5.1)	5 (3.7)	6 (7.1)	0.057^#^
Diploma/Certificate	11 (9.4)	16 (11.8)	16 (19.0)	
Associate Degree/Higher Diploma	17 (14.5)	20 (14.7)	14 (16.7)	
Bachelor’s degree or above	83 (70.9)	95 (69.9)	48 (57.1)	
*Independent variables*				
Participation in gambling activities, Median (IQR)	1 (0–2)	1 (0–2)	1 (1–2)	0.001 (1/0.001/0.003)^#^
Motives of gacha purchase, Median (IQR)	2 (1–3)	3 (2–4)	4 (3–6)	<0.001 (<0.001/<0.001 < 0.001)^#^
**Daily time spent on gacha game, n (%)**				
0–3 h	94 (80.3)	84 (61.8)	38 (45.2)	<0.001 (0.002/<0.001/0.013)^#^
4–6 h	19 (16.2)	40 (29.4)	35 (41.7)	
7–9 h	3 (2.6)	9 (6.6)	6 (7.1)	
≥10 h	1 (0.9)	3 (2.2)	5 (6.0)	
**Monthly expenses on gacha purchase, n (%)**				
≤ USD$50	109 (93.2)	101 (74.3)	30 (35.7)	<0.001 (0.004/<0.001/<0.001)^#^
USD$51-100	7 (6.0)	19 (14.0)	20 (23.8)	
USD$101-200	0 (0)	11 (8.1)	13 (15.5)	
> or = USD201	1 (0.9)	5 (3.7)	21 (25)	
**DASS-21-C subscales, Median (IQR)**				
Depression score	4 (0–10)	10 (2–16)	16 (9–28)	<0.001 (0.003/<0.001 < 0.001)^#^
Anxiety score	2 (0–6)	4 (2–8)	13 (5–22)	<0.001 (0.012/<0.001 < 0.001)^#^
Stress score	4 (0–12)	10 (4–14)	18 (12–28)	<0.001 (0.005/<0.001 < 0.001)^#^

M, mean; SD, standard deviation; IQR, Interquartile range; DASS-21-C, Depression, Anxiety, Stress 21 – Chinese version. a: *p* value between non/low-risk of problem gambler and moderate-risk of problem gambler. b: *p* value between non/low-risk of problem gambler and high-risk of problem gambler. c: *p* value between moderate-risk of problem gambler and high-risk of problem gambler. *Chi-squared test. ∧One-way ANOVA. ^#^Kruskal Wallis One-way ANOVA.

Table 2 describes the motives for gacha purchase. The study found that all motives were significantly different among the three groups except for “no specific purposes” and “support income of gaming companies.” High-risk problem gamblers exhibited the highest percentages in the following motives: felt excited (69%), gained social recognition (63.1%), obtained what friends had had (59.5%), sped up the game progress (59.5%), obtained favorite virtual items (91.7%), and seized the time-limited offer (61.9%).

**TABLE 2:** Comparison of motives for gacha purchase among three levels of problem gambling.

Motives	Non/low-risk problem gambler (*n* = 117)	Moderate-risk problem gambler (*n* = 136)	High-risk problem gambler (*n* = 84)	*P*-value
**No specific purposes, n(%)**				
Yes	15 (12.8)	20 (14.7)	16 (19.0)	0.470
No	102 (87.2)	116 (85.3)	68 (81.0)	
**Feel relaxed, n(%)**				
Yes	7 (6.0)	20 (14.7)	26 (31.0)	<0.001*
No	110 (94.0)	116 (85.3)	58 (69.0)	
**Feel excited, n(%)**				
Yes	18 (15.4)	48 (35.3)	43 (51.2)	<0.001*
No	99 (84.6)	88 (64.7)	41 (49.8)	
**Seize time-limited offers, n(%)**				
Yes	47 (40.2)	71 (52.2)	52 (61.9)	0.009**
No	70 (59.8)	65 (47.8)	32 (38.1)	
**Gaining social recognition, n(%)**				
Yes	16 (13.7)	35 (25.7)	53 (63.1)	<0.001*
No	101 (86.3)	101 (74.3)	31 (36.69)	
**Want what my friends have, n()%**				
Yes	8 (6.8)	15 (11.0)	50 (59.5)	<0.001*
No	109 (93.2)	121 (89.0)	34 (40.5)	
**Speed up the game progress, n(%)**				
Yes	40 (34.2)	57 (41.9)	50 (59.5)	0.001**
No	77 (65.8)	79 (58.1)	34 (40.5)	
**Obtain favourite items (e.g. costume, weapon), n(%)**				
Yes	89 (76.1)	115 (84.6)	77 (91.7)	0.012***
No	28 (23.9)	21 (15.4)	7 (8.3)	
**Support income of gaming companies, n(%)**				
Yes	30 (25.6)	45 (33.1)	27 (32.1)	0.399
No	87 (74.4)	91 (66.9)	57 (67.9)	

*p < 0.001; **p < 0.01; ***p < 0.05.

The total PGSI score was positively correlated with age (*r* = 0.196, *p* < 0.001); daily time spent on gacha purchase (*r* = 0.259, *p* < 0.001); monthly expenses for gacha purchase (*r* = 0.478, *p* < 0.001); participation in gambling activities (*r* = 0.168, *p* = 0.002); motives for gacha purchase (*r* = 0.465, *p* < 0.001); and stress, anxiety, and depression subscale scores (*r* = 0.496, 0.437, and 0.337, *p* < 0.001, respectively). Only educational level was negatively correlated with the total PGSI score (*r* = -0.098, *p* < 0.05). Table 3 presents the correlations among the variables.

**TABLE 3:** Correlations between total PGSI score and study variables (*N* = 337).

	Total PGSI score	
	*r*	*P*-value
Age	0.196	< 0.001*
Educational level	–0.098	0.045***
Daily time spent on gacha games	0.259	< 0.001*
Monthly expenses on gacha purchase	0.478	< 0.001*
DASS - stress score	0.496	< 0.001*
DASS - anxiety score	0.437	< 0.001*
DASS - depression score	0.337	< 0.001*
Participation in gambling activities	0.168	0.002**
Motives for gacha purchase	0.465	< 0.001*

DASS, Depression, Anxiety, Stress 21; PGSI, Problem Gambling Severity Index. *p < 0.001; **p < 0.01; ***p < 0.05.

### Relationship among risk of problem gambling, demographics, gaming behavior, and psychological distress

Table 4 summarizes the regression model for predicting the risk of problem gambling with demographics and other study variables. The R-squared value (*R*^2^ = 0.521) for the regression was significantly different from 0 [*F*(5,331) = 71.895, *p* < 0.001]. The overall model accounted for 52.1% of the variance of total PGSI scores, thus suggesting that 52.1% of the variance of the total PGSI score could be explained by the following five variables in the model: stress (16% unique variance), monthly expenses on gacha purchase (11.5% unique variance), number of gacha purchase motives (8.9% unique variance), anxiety (12.6% unique variance), and number of gambling activities participated (2.8% unique variance). The B coefficients showed that all five independent variables were significant predictors with none of the 95% confidence intervals being across 0. The constructed model thus suggests that gacha gamers who experience greater stress and anxiety, spend more on gacha purchases, have more motives for gacha purchases; and participate in more types of gambling activities have a tendency to be problem gamblers.

**TABLE 4:** Predictors of the total PGSI score modelled by stepwise multiple regression (*N* = 337).

Predictor	Standardized coefficient (β)	Unstandardized coefficient (β)	*t*	*P*-value
DASS - stress score	0.272	0.039	3.818	< 0.001*
DASS - anxiety score	0.224	0.046	3.210	0.001**
Monthly expenses on gacha purchase	0.265	0.250	6.283	< 0.001*
Motives of gacha purchase	0.189	0.132	4.258	< 0.001*
Participation in gambling activities	0.117	0.199	2.961	0.003**
*Model statistics*				
R^2^			0.521	
Adjusted R^2^			0.513	
R			0.722	
F			71.895	< 0.001*

DASS, Depression, Anxiety, Stress 21. *p < 0.001; **p < 0.01.

## Discussion

### Gacha gaming behavior and risk of problem gambling

The current study found that nearly 80% of high-risk problem gamblers were female, which is more than double the percentage of females in the moderate-risk group. This is inconsistent with the extant literature, which largely presents male gamers as having higher problem gambling risk than their female counterparts (15, 25, 45). The incongruent findings between present and previous studies could be explained by the pandemic situation in Hong Kong during the period of data collection. Recent evidence has shown that females have higher stress levels and were more likely to have depression and anxiety compared to males during the COVID-19 pandemic (59–62). Also, psychological distress is one of the factors reported to be associated with problem gambling in females (63). Therefore, the pandemic-induced stress and other psychological distress may make female participants more susceptible to problem gambling. As the percentage of female mobile gamers has been progressively increasing over the past ten years, i.e., from 38 to 46%, it is foreseeable that more female gamers may engage in mobile games with microtransactions (64). Accordingly, healthcare providers must pay special attention to female gamers who are under stressful situations and offer preventive measures such as fostering their coping efficacy to improve their psychological well-being (59).

The present study found that high-risk problem gamblers did spend significantly more time on gaming compared to the non/low-risk and moderate-risk groups. Nearly 55% of high-risk problem gamblers spent four or more hours (equivalent to ≥28 h/week) playing gacha games every day, which exceeds the duration reported by King et al. for gamers playing Fortnite (a kind of Freemium game) with a weekly playtime of 21.4 h (65). Although daily time spent on gacha games is not a significant predictor for problem gambling, this finding may alert healthcare professionals to the fact that gacha gamers may have problem gambling issues if they spend 4 h or more on gaming each day. It is also consistent with a local survey that over 50% of gacha gamers spent 3 h or above on gaming every day (9). Recent statistics (66) showed that Asian gamers spend much more time on gaming compared to the global weekly gaming time for all individuals, regardless of geography (8.45 h/week). China was ranked the first (12.69 h/week), followed by Vietnam (10.16 h/week) and India (8.61 h/week). Asian gamers, especially Chinese, are therefore at the greatest risk of problem gambling. The risk of becoming a problem gambler may have further increased during the pandemic given that people’s video game playtimes during the pandemic increased by 42% in the Asia-Pacific region (67). One point worthy noting is that the average daily hours spent gaming in the present study was much higher than those reported in other studies. The reasons for that are (1) the present study reported the daily hours spent on gaming according to the three risk levels of problem gambling but did not mix them together; (2) the study focused on time spent playing gacha games but not all kinds of video games; and (3) the study was conducted at the peaks of the COVID-19 pandemic in Hong Kong, during which all schools, including university/tertiary institutions, either switched to online learning or temporarily suspended classes. In addition, many economic activities were partially or totally suspended. More free time at home may have resulted in spending more time on video gaming in the young adult population.

### Relationship between risk of problem gambling, gaming behavior, and psychological distress

Unlike time spent on gacha games, monthly expenses on gacha purchases and motives are positively associated with the risk of problem gambling. This suggests that gamers would have a higher risk of problem gambling if they spend more on gacha games and have more motives for gacha purchases. The positive association between spending on gacha purchase and risk of gambling is supported by previous studies for loot box (33, 36, 40, 68). Garea et al. found a positive correlation between loot box spending and gambling symptoms (36). Drummond et al. found that the association between loot box spending and problem gambling symptoms was significant (40). The motives that were found to exist in significantly higher proportions in the high-risk group than in the other two groups are related to social recognition and stress relief, such as feeling relaxed, gaining social recognition, and want what their peers have. Similar motives were identified in Zendle et al. (18). This finding is also supported by previous studies. In a study of Swedish adolescents, Hellostrom et al. (69), found that gaming for escape, gain social status, or to pacify others rather than time spent gaming was associated with an increased risk of problem gambling and other negative consequences. Lelonek-Kuleta and Bartczuk found that an escape-type coping strategy was associated with a higher risk of gambling disorders in E-sport gamers (70).

Approximately 65% of the participants in the high-risk group reported spending USD $51 or more every month on gacha purchases, among which 25% had spent an astonishing more than USD $200 per month. It is similar to the local report that about 75% participants with an average of 26.8 years spent USD$125 or below for gacha purchase under COVID-19 pandemic (9). The significantly higher spending on gacha in the high-risk group may be attributable to their intention to gain recognition among peers or cope with stress (27, 31, 71). Using distractions such as gaming to cope with stress is an emotion-focused coping strategy. If gacha gamers are used to adopting emotion-focused strategies to cope with stress, they may have a higher problem gambling risk. Furthermore, those categorized as high sensation seekers are also more likely to become problem gamblers as a significantly higher proportion of those in the high-risk group reported feeling excited as a factor compared to the other two groups. Stress and anxiety are another two factors that significantly contributed to predicting the risk of problem gambling. Their positive association indicates that higher stress and anxiety levels would lead to higher risk of problem gambling, which is congruent with previous studies related to loot box (35, 41–43, 68). In response, healthcare professionals should focus on building up gacha gamers’ internal and external resources to manage stress so as to consolidate their resilience against stressful situations or environments. In addition, a proper stress management approach could reduce gamers’ stress and anxiety. It is especially important in these last few years as the COVID-19 pandemic has greatly hampered normal economic activities and people’s daily lives. The World Health Organization recently published a scientific brief presenting the impacts of the COVID-19 pandemic on mental health worldwide. Anxiety disorders were found to have increased by about 25.6% under the COVID-19 pandemic (62). By age, young adults those aged 20–24 years were more affected than older adults (62). These statistics echo those of the present findings in which 25% of the study participants (all in the high-risk group) were found to have moderate stress, anxiety, and depression. Seemingly, the COVID-19 pandemic has greatly affected young adults’ psychological well-being. Accordingly, devising preventive measures targeted at gacha gamers who possess more risk factors of problem gambling belongs at the top of the agenda for mental health professionals, particularly given that there is still a long way to go before the COVID-19 pandemic ends.

### Limitations

This study has several limitations. First, data was collected through a self-report survey. Consequently, although face validity and test-retest reliability were performed with satisfactory results prior to the main study, the collected data may be susceptible to social desirability bias. Second, the data collection was confined to Hong Kong. The demographic characteristics of young adults and the pandemic situation in Hong Kong may differ from those in Mainland China. Therefore, the results may not be generalizable to the context of Mainland China. Third, the predictive model was established with cross-sectional data. Causal relationship between the dependent and independent variables cannot be drawn. To verify the present model and widen the generalizability of the results, future studies should adopt a longitudinal approach. Sampling should be carried out in different provinces of Mainland China or even other Asian countries.

### Practical implications

To the best of the researchers’ knowledge, previous studies largely focus on problem gambling among gamers participating in loot box purchases but not in gacha purchases. As gacha is the predominant form of video game microtransaction in the Asian gaming market, the research team initiated the present study to examine the association between problem gambling and gacha purchase in Chinese gamers. It is hoped that this study fills the gap in the existing body of knowledge related to problem gambling and video game microtransactions, especially pertaining to gacha. From a primary healthcare perspective, early identification of potentially high-risk gacha gamers can prevent persistent inappropriate in-game purchase behavior that may lead to gambling disorders. The results of the present study will add insights into the direction of preventing problem gambling in gacha gamers. From a research perspective, the model constructed in the present study might guide the direction of future gambling studies related to video game microtransaction.

## Conclusion

The constructed regression model demonstrated that stress, anxiety, expenses on gacha purchases, number of motives for gacha purchase, and participation in gaming activities are five key contributing factors that can be utilized to predict problem gambling risk in young adult gacha gamers. Young adult gacha gamers experiencing greater stress and anxiety tend to spend more on gacha purchases, have more motives for gacha purchase, and participate in more gambling activities. This group is at a particularly higher risk of becoming problem gamblers. Additionally, the present study found a significantly higher proportion of female gamers exists in the high-risk problem gambling group, indicating that female gamers are inclined to be problem gamblers, especially under COVID-19 pandemic, than male gamers because females were more emotionally affected by the pandemic and suffered more stress and anxiety. The study findings shed light on the risk factors of becoming a problem gambler among young adult gacha gamers. The five contributing factors in the constructed model could facilitate stakeholders’ identification of young adult gacha gamers at risk of problem gambling in the community. Furthermore, social and healthcare professionals can develop targeted health education and promotion interventions, particularly those aimed at stress management and encouraging positive coping measures for those vulnerable gacha gamers before they become pathological gamblers. Further studies are needed to verify the causal relationships established in the constructed model using a longitudinal approach.

## Data availability statement

The raw data supporting the conclusions of this article will be made available by the authors, without undue reservation.

## Ethics statement

The studies involving human participants were reviewed and approved by Ethics Review Committee, Tung Wah College. Written informed consent for participation was not required for this study in accordance with the national legislation and the institutional requirements.

## Author contributions

AT and RL conceptualized the study. AT, PL, and SL drafted the manuscript and analyzed the data. SS, CY, AT, and SL collected the data. All authors were involved in revising the manuscript and approved the final version.
